# Anti-*Candida* Activity of Hyaluronic Acid Combined with *Lactobacillus* *crispatus* Lyophilised Supernatant: A New Antifungal Strategy

**DOI:** 10.3390/antibiotics10060628

**Published:** 2021-05-25

**Authors:** Carola Parolin, Angela Abruzzo, Barbara Giordani, Josidel C. Oliver, Antonella Marangoni, Barbara Luppi, Beatrice Vitali

**Affiliations:** 1 Department of Pharmacy and Biotechnology, Alma Mater Studiorum–University of Bologna, Via San Donato 19/2, 40127 Bologna, Italy; carola.parolin@unibo.it (C.P.); angela.abruzzo2@unibo.it (A.A.); barbara.giordani4@unibo.it (B.G.); jsdl.oliver@gmail.com (J.C.O.); barbara.luppi@unibo.it (B.L.); 2 Department of Microbiology and Immunology, Federal University of Alfenas, Alfenas 37130-001, Brazil; 3 Microbiology, Department of Experimental, Diagnostic and Specialty Medicine, Alma Mater Studiorum–University of Bologna, Via Massarenti 9, 40138 Bologna, Italy; antonella.marangoni@unibo.it

**Keywords:** vulvovaginal candidiasis, vaginal lactobacilli, hyaluronic acid, *Lactobacillus* *crispatus*, *Candida*

## Abstract

Vulvovaginal candidiasis (VVC) and recurrencies are common in reproductive-aged women. The emergence of *Candida* strains resistant to conventional antimycotic drugs prompted the search for alternative therapies. Hyaluronic acid (HA), a uniform and linear glycosaminoglycan, has been proposed as an anti-*Candida* agent. Vaginal lactobacilli and their derivatives, including cell free culture supernatants (CFS), represent potential strategies for the treatment of *Candida* infections. In the present paper, the anti-*Candida* potential of HA and lyophilised CFS (LCFS), obtained from the vaginal strain *Lactobacillus crispatus* BC5, was investigated. HA and LCFS proved to be active towards a panel of clinical *Candida* isolates belonging to different species in a dose dependent manner and their association maintained the antifungal activity. Notably, also *Candida* species generally resistant to conventional antifungals resulted sensitive. A vaginal matrix based on microcrystalline cellulose and containing effective doses of both agents was developed and characterised. This vaginal formulation showed mucoadhesive ability and almost abrogated *Candida albicans* growth. In conclusion, HA and LCFS from *L. crispatus* BC5 are thus good candidates to design a new therapeutic strategy to counteract VVC, and the proposed vaginal matrix represents a promising prototype.

## 1. Introduction

*Candida* species are common commensals of the human body mucosae, especially colonizing gastrointestinal, respiratory and genitourinary tracts. In particular conditions not yet fully understood, *Candida* spp. could become robust opportunistic pathogens, and can give rise, in women, to symptomatic vulvovaginal candidiasis (VVC), compromising their quality of life. Indeed, VVC affects 75% of reproductive-aged women at least once during their lives, with about 5% of them suffering recurrences; *Candida albicans* is considered the major responsible of VVC cases, although in the last years different species, such as *Candida glabrata*, *Candida krusei* and *Candida tropicalis,* have emerged [1]. Topic and oral antifungals, especially azoles, are conventionally employed for VVC treatment; however, the prolonged exposition to these drugs can promote the appearance of resistant *Candida* strains. This phenomenon is particularly frequent for *Candida* isolates belonging to non-*albicans* species as, for example, *C. glabrata* and *C. krusei* [2], which are more difficult to eradicate. Thus, the search for new anti-*Candida* agents and the development of new pharmacological strategies are required to face these types of infections [3,4].

Hyaluronic acid (HA) is a uniform, linear glycosaminoglycan, composed of repeated disaccharide units that can reach high length and molecular weight. HA is abundant in skin and connective tissues and represents one of the main components of the extracellular matrix, where numerous HA polymers can organise in reticular structures. Due to its hygroscopic property, biocompatibility and biodegradability, HA is widely used in medicine and in pharmaceutical formulations. Antiviral activity of high molecular weight HA has been reported [5], and antibacterial and antifungal activity towards a few *Candida* strains has been proposed [6,7].

In our previous studies we demonstrated the fungistatic and fungicidal activity of cell-free culture supernatants of vagina-derived *Lactobacillus* strains towards a panel of *Candida* vaginal isolates, suggesting a potential new strategy for the treatment of *Candida* infections, especially at the local level [8]. Among the tested vaginal *Lactobacillus* strains, *L. crispatus* BC5 showed a broad inhibitory activity towards *Candida* isolates, including *C. albicans*, *C. tropicalis* and *Candida lusitaniae.*

In the present study, the anti-*Candida* activity of high molecular weight HA was tested against vaginal clinical isolates, and HA was employed alone or in combination with *L. crispatus* BC5 cell-free culture supernatant, after lyophilization (LCFS). In addition, a matrix based on microcrystalline cellulose, containing effective doses of HA and LCFS was developed, characterized in terms of technological properties and evaluated for its activity against *C. albicans* growth.

## 2. Results

### 2.1. HA Reduced Candida Growth

**Figure 1:**
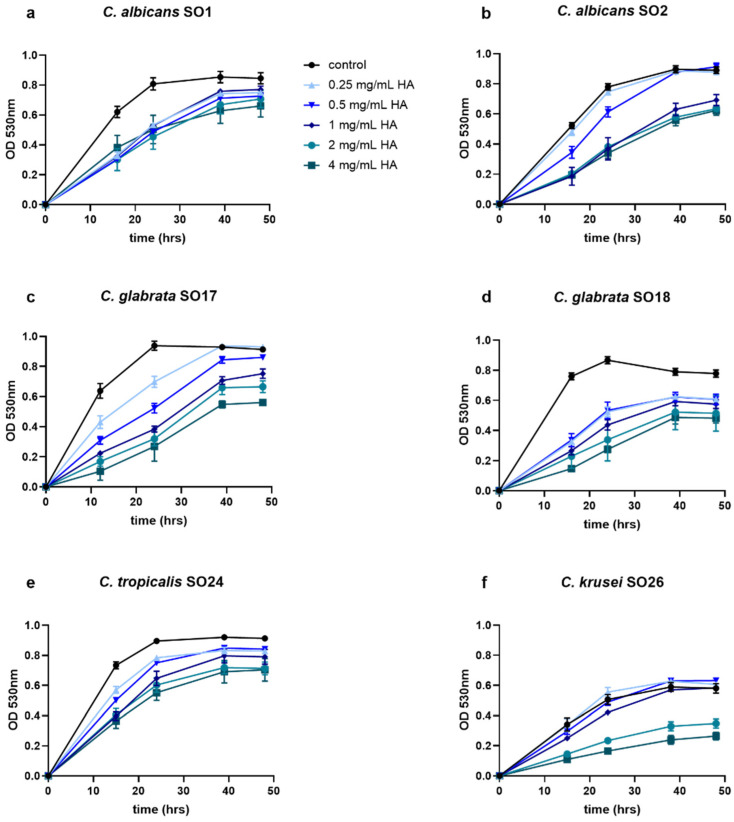
Growth curves of (**a**) *C. albicans* SO1, (**b**) *C. albicans* SO2, (**c**) *C. glabrata* SO17, (**d**) *C. glabrata* SO18, (**e**) *C. tropicalis* SO24, and (**f**) *C. krusei* SO26 in the presence of different concentrations of HA (0.25–4 mg/mL).

Vaginal clinical isolates of *Candida* spp. were tested for their susceptibility to HA, measuring the effect of different doses of HA on *Candida* growth over time. All the tested strains were susceptible to HA and the anti-*Candida* activity was dose-dependent (Figure 1). Specifically, *C. albicans* SO1, *C. glabrata* SO17, *C. glabrata* SO18 and *C. tropicalis* SO24 resulted susceptible even to the lowest dose of HA (0.25 mg/mL), since growth data were significantly different from their respective controls (ANOVA, *p* < 0.05). *C. albicans* SO2 and *C. krusei* SO26 showed a significant reduction of growth at concentrations above 1 and 2 mg/mL of HA, respectively.

### 2.2. Anti-Candida Effect of Combined HA-LCFS

**Figure 2:**
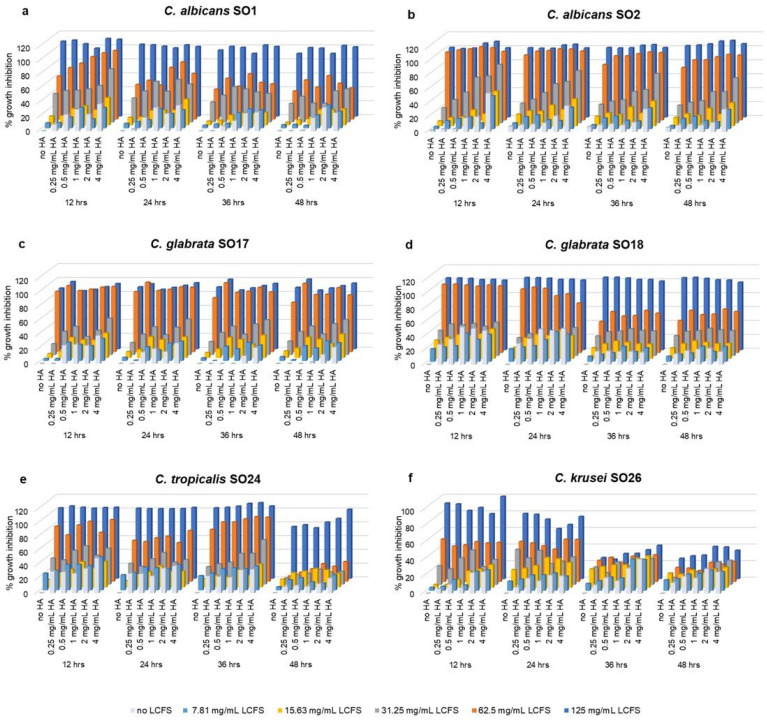
Growth inhibition (%) of (**a**) *C. albicans* SO1, (**b**) *C. albicans* SO2, (**c**) *C. glabrata* SO17, (**d**) *C. glabrata* SO18, (**e**) *C. tropicalis* SO24, and (**f**) *C. krusei* SO26 in the presence of different combinations of LCFS (7.81–125 mg/mL) and HA (0.25–4 mg/mL) over time.

The same concentrations of HA were tested in combination with different doses of LCFS, ranging from 7.81 to 125 mg/mL towards *Candida* isolates, growth inhibition was calculated and reported in Figure 2. Regarding LCFS alone, it resulted effective towards all tested *Candida* strains. LCFS minimum inhibitory concentration (MIC) that inhibits 50% of growth (calculated at 24 h) was equal to 62.5 mg/mL for all *Candida* strains, with the only exception of *C. krusei* SO26, which showed a MIC of 125 mg/mL. The highest tested concentration (125 mg/mL) totally inhibited *Candida* growth at 12 h and 24 h, and a marked inhibition (above 75%) persisted over 48 h, except for *C. krusei* SO26. When LCFS was added with different doses of HA, its antifungal activity was maintained and, in some cases, slightly enhanced for high concentrations of HA (2 and 4 mg/mL) at 12 and 24 h. As an example, for *C. albicans* SO2 treated with 15.63 mg/mL LCFS, a clear HA dose-dependent profile was registered. However, fractional inhibitory concentration index (FICI) values were calculated for each *Candida* isolate on data set registered at 24 h of treatment and did not demonstrate a synergic effect of HA and LCFS (Table 1). Only for *C. albicans* isolates, an additive effect was observed (FICI values equal to 1 and 0.75 for *C. albicans* SO1 and *C. albicans* SO2, respectively). As the combination of HA 2 mg/mL and LCFS 62.5 mg/mL inhibits more than 50% of *Candida* growth of all the tested strains at early time points, it was chosen for the formulation of a matrix intended for vaginal application.

### 2.3. Characterization of Vaginal Matrices

Matrices containing HA and LCFS were easily taken out of blisters and they did not chip, crack, or crumble when handled during the subsequent studies and use. Matrices showed cylindrical shape, a mean thickness of 4.07 ± 1.19 mm and a mean diameter of 12.96 ± 0.43 mm. In-vitro residence time measurements demonstrated that the matrices containing HA and LCFS remained attached to the porcine vaginal mucosa for 125 ± 9 min, when they completely disintegrated favoring the release of the active substances. On the contrary, the time needed for the detachment of control matrices (based only on MCC) from the same mucosa was lower than 1 min.

### 2.4. Vaginal Matrices Reduce C. albicans Growth

The vaginal matrices were tested towards *Candida* growth in order to investigate whether HA and LCFS, formulated with microcrystalline cellulose (MCC), retained their biological activity. *C. albicans* SO1 was taken as a model test strain. Considering the volume of vaginal fluid (0.6–0.8 mL recovered in 45 min) [9] a volume equal to 2 mL was used in the test. Thus, each matrix was put in a volume of 2 mL of *Candida* suspension in SD broth, allowing to obtain the final concentration of 2 mg/mL of HA and 62.5 mg/mL of LCFS after dissolution. In the presence of a MCC matrix containing HA and LCFS, a marked decrease of fungal growth was observed; in particular, *C. albicans* SO1 growth was reduced by 89.1% (6 h) and 81.4% (24 h) with respect to the system without matrix, and by 83.3% (6 h) and 81.5% (24 h) with respect to MCC matrix (Table 2). Such slight decrease of inhibition over time suggested that HA/LCFS matrix activity is more evident at very early time points. As expected, matrices prepared with only MCC did not significantly affect *C. albicans* SO1 growth.

## 3. Discussion

The need of new therapeutic strategies to counteract VVC and recurrencies raised from the growing emergence of *Candida* strains resistant to conventional antimycotic drugs. In this regard, HA shows a double advantage: (i) fungistatic activity described in some studies [6,10] and (ii) hydration ability and mucoadhesive properties that can be exploited in the development of new pharmaceutical formulations [11]. Besides this, probiotic strains, including vaginal lactobacilli, and their derivatives represent promising candidates to face VVC for their natural antimicrobial potential [4]. Many members of the *Lactobacillus* genus, such as *L. pentosus*, *L. rhamnosus*, *L. paracasei*, and *L. delbrueckii*, have been recognised as probiotics and produce compounds with antifungal activity. Among *Lactobacillus* derivatives, diverse organic acids, active biomolecules, bacteriocins or similar can impair *Candida* growth, adhesion and biofilm formation, or act as immunomodulators [4,12]. Soluble bioactive compounds can be secreted by probiotics cells and thus retrieved in their culture supernatants. In our previous researches we identified various *L. crispatus* derivatives active towards *Candida* spp. [8,13,14]. In particular, culture supernatants produced by *L. cripatus* BC5 vaginal strain showed a broad growth inhibitory activity towards *Candida* isolates [8]; moreover *L. crispatus* BC5 cells, together with the prebiotic fructo-oligosaccharide and the antioxidant agent ascorbic acid, have been included in a symbiotic vaginal insert, for the prophylaxis and therapy of fungal and bacterial vaginal infections [15]. In the present work, inhibitory activity of HA has been demonstrated towards *Candida* clinical isolates belonging to different species, and its use in combination with a lyophilized form of *L. cripatus* BC5- derived culture supernatants (LCFS) has been proposed. The association of HA with LCFS showed additive effects against *C. albicans* isolates, while for the other tested *Candida* isolates LCFS retained its antifungal activity in the presence of HA. Notably, *C. glabrata* isolates were sensitive to both LCFS and HA, although this species is often reported as resistant to conventional antifungal drugs [16]. *C. krusei* SO26 resulted to be the less sensitive isolate in our study, as it showed the highest values of HA and LCFS MIC and the growth reduction run out after 24 h.

In order to assure accurate dosing of both LCFS and HA, solid dosage forms removable from their packaging and deliverable in the vaginal cavity, without mass loss, were developed. Lyophilization of a homogeneous dispersion composed of LCFS, HA and MCC allowed to obtain uniform matrices with good handling properties. 

The ability of the matrix to adhere to the mucosa is of particular interest in order to overcome its rapid removal from the vaginal cavity, thus favoring the presence of LCFS and assuring its efficacy for a longer period. Considering that matrices based only on MCC did not display any ability to interact with vaginal mucosa (residence time lower than 1 min), the mucoadhesive properties of the proposed matrix (residence time of about 125 min) could be attributed to the presence of HA [17], able to establish hydrogen bonds with the mucin chains, and LCFS, composed of sugars, amino acids, and lipid molecules able to promote the matrix hydration and its subsequent adhesion to mucus [15]. The individual contribution of each substance to the mucoadhesion was not determined in this study, as it was not our aim to deliver HA and LCFS as separate active agents. 

The MCC matrix containing HA and LCFS showed a marked anti-*Candida* effect, almost abrogating *C. albicans* growth after 6 and 24 h of contact. HA/LCFS activity was thus retained in the final formulation, which is able to adhere to the mucosa and to successfully release the active agents. Nevertheless, the observed slight decrease of inhibition over time could be overcome by an appropriate multiple dose regimen. Further studies are needed to better explore the in-vivo permanence of the matrices in the vaginal cavity and to optimize the release of the active compounds over time and the administration regimen.

## 4. Materials and Methods

### 4.1. Candida Strains and Culture Conditions

*Candida* strains used in the present study were isolated from vaginal swabs of premenopausal, VVC affected women, submitted to the Microbiology Laboratory of Sant’Orsola-Malpighi University Hospital of Bologna (Italy) during routine diagnostic procedures. Isolates were coded to assure full anonymousness and belong to the laboratory collection. Specifically, *C. albicans* SO1, *C. albicans* SO2, *C. glabrata* SO17, *C. glabrata* SO18, *C. tropicalis* SO24, and *C. krusei* SO26 were employed [14]. *Candida* isolates were grown aerobically at 35 °C for 24–48 h in Sabouraud dextrose (SD) agar (Beckton, Dickinson and Co., Milan, Italy).

### 4.2. Lactobacillus Cultivation and Preparation of LCFS

*L. crispatus* BC5 was previously isolated from the vaginal swab of a healthy premenopausal woman, following the protocol approved by the Ethics Committee of the University of Bologna (52/2014/U/Tess) [8]. The strain was cultured in de Man, Rogosa and Sharpe (MRS) (Beckton, Dickinson and Co., Milan, Italy) broth supplemented with 0.05% L-cysteine (Sigma-Aldrich, Milan, Italy), at 37 °C in anaerobic jars containing GasPak EZ (Beckton, Dickinson and Co., Milan, Italy). 100 mL of an overnight culture were inoculated in 900 mL of MRS broth and allowed to grow for additional 24 h in anaerobic jars. The bacterial suspension was centrifuged at 10.000× *g* for 10 min and the supernatant filtered through a 0.22 μm pore size filter (PES 0.22 μm syringe filters, VWR International, Milan, Italy). Cell-free supernatant was then lyophilised at 0.01 atm and −45 °C (Christ Freeze Dryer ALPHA 1–2, Milan, Italy), obtaining lyophilised cell free supernatant (LCFS). Analogously, lyophilised MRS medium (LMRS) was prepared. LCFS and LMRS were stored at −20 °C until used.

### 4.3. HA Susceptibility Assays

Hyaluronic acid (HA, MW 1800–2300 kDa, Farmalabor srl, Canosa di Puglia, Italy) was suspended in sterile supplemented RMPI 1640 medium (2% *w/v* glucose, 0.165 mol/L 3-(N-morpholino) propanesulfonic acid (MOPS), pH 7.0, all purchased from Merck, Milan, Italy) to obtain a final concentration of 8 mg/mL and stirred for 24 h to obtain complete dissolution. HA solution was kept at −20 °C until used. The assay was performed in 96-wells microplates in accordance with the European Committee on Antimicrobial Susceptibility Testing (EUCAST) guidelines [18]. Briefly, each well of the microplate was filled with 100 µL of *Candida* suspensions (1–5 × 10^5^ CFU/mL, prepared in supplemented RPMI 1640) added with 100 µL of HA dilutions in supplemented RPMI 1640. The final concentrations of 0.25, 0.5, 1, 2 and 4 mg/mL of HA were tested; *Candida* inoculated in supplemented RPMI 1640 alone was used as positive growth control. The plates were incubated at 35 °C and *Candida* growth was followed by measuring the optical density at 530 nm (OD_530_) by a multiplate reader (EnSpire multimode plate reader, Perkin-Elmer, Milan, Italy) every approximately 12 h for 48 h. Each condition was tested in quadruplicate, each *Candida* strain was tested in at least two independent experiments.

### 4.4. HA-LCFS Susceptibility Assays

HA was suspended in sterile supplemented RMPI 1640 medium at a concentration of 8.8 mg/mL; LCFS or LMRS were suspended in sterile supplemented RMPI 1640 medium at a concentration of 0.5 g/mL. The assay was performed in 96-wells microplates in accordance with EUCAST guidelines, as described above. Briefly, each well of the microplate was filled with 100 µL of *Candida* suspensions (1–5 × 10^5^ CFU/mL) containing different concentrations of HA, added with 100 µL of LCFS dilutions. The final concentrations of 7.81, 15.63, 31.25, 62.5 and 125 mg/mL LCFS, in combination with 0.25, 0.5, 1, 2 and 4 mg/mL HA were tested; *Candida* inoculated in 7.81, 15.63, 31.25, 62.5 and 125 mg/mL LMRS was used as positive growth control. The plates were incubated and *Candida* growth followed as described above. At each time point, *Candida* growth inhibition (%) was calculated as follows:

growth inhibition (%) = [1 − (OD_530_ sample/OD_530_ positive growth control)] × 100

Each condition was tested in triplicate, each *Candida* strain was tested in at least two independent experiments. The antifungal minimum inhibitory concentration (MIC) is defined as the lowest concentration of HA or LCFS (or combination) giving rise to ≥50% inhibition of growth compared to the respective positive control, in accordance with EUCAST guidelines [18]. Values that were above or equal to the maximum concentration tested were transformed to equal. Data obtained at 24 h of treatment were analysed using the model-fractional inhibitory concentration index (FICI) method based on the Loewe additivity theory, as reported in Marangoni et al. (2017) [19]. FICI was calculated as follows:

FICI = FIC (HA) + FIC (LCFS)

where

FIC (HA) = MIC of the combination/MIC of HA alone
FIC (LCFS) = MIC of the combination/MIC of LCFS alone
FICI values ≤ 0.5 indicated a synergic effect, while antagonism was defined as a FICI value > 4, addition was defined as a FICI value in the range 0.5–1. A FICI value between 1 and 4 was considered indifferent.

### 4.5. Preparation and Characterization of Vaginal Matrices

Matrices containing HA and LCSF were produced by lyophilisation in the presence of microcrystalline cellulose (MCC, Farmalabor srl, Canosa di Puglia, Italy), a widely used diluent for the preparation of solid dosage forms. Firstly, HA was dissolved under stirring (200 rpm) in demineralized water at room temperature for 24 h. Then, MCC was added to the HA solution and stirred (50 rpm) at room temperature for 12 h, thus obtaining a dispersion with final concentrations of HA and MCC of 0.8% *w*/*w* and 4% *w*/*w*, respectively (weight ratio MCC/HA 5:1). Subsequently, an adequate volume of LCFS solution (obtaining dissolving LCFS in water under stirring for 1 h; 54.23% *w*/*w*) was added to the dispersion in order to obtain a final concentration equal to 25% *w*/*w*. Approximately 0.5 g of the dispersion (containing 4 mg of HA and 125 mg of LCFS) were placed into each cavity (diameter 13 mm) of a blister pack (Farmalabor srl, Canosa di Puglia, Italy), frozen overnight at −20 °C and lyophilised. Matrices were stored at −20 °C until use. Matrices containing only MCC were also produced as control formulations for mucoadhesion and antimicrobial studies. Matrices were weighted and measured for diameter and thickness through an electronic digital caliper (art. 1367 E 2900, Shanghai ShangErBo Import & Export Co., Shanghai, China). Residence time measurements allowed the evaluation of the mucoadhesive strength as well as disintegration characteristics of the matrices. For this study, porcine vaginal mucosa was used due to its similarity to the human vaginal tissue [20] and isolated as previously described [7]. The mucosa was hydrated for 15 min with 0.2 mL of mucin (Sigma-Aldrich, Milan, Italy) suspension (0.5% *w*/*v*) in 0.1 M KH_2_PO_4_ buffer at pH 4.5, simulating human vaginal pH [21]. Indeed, matrix was attached to the mucosa with a slight pressure for 15 s and immersed inside a beaker containing 10 mL of phosphate buffer at pH 4.5. The time taken by the matrix to completely detach from the mucosa was considered as the residence time. All the measurements were performed in triplicate.

### 4.6. Vaginal Matrices Anti-Candida Activity

Vaginal matrices were tested towards *C. albicans* SO1 growth. *C. albicans* SO1 was inoculated in 2 mL of SD broth at 1–5 × 10^5^ CFU/mL, added with a vaginal matrix and incubated at 35 °C for 6 h and 24 h, with gentle agitation (50 rpm). MCC matrices containing HA and LCSF were compared with matrices containing only MCC. Antifungal activity was analysed by evaluating *C. albicans* viability (CFU/mL) over time. *Candida* suspensions were opportunely diluted in sterile saline then plated onto SD agar plates. Plates were incubated at 35 °C for 24–48 h. Each condition was tested in triplicate in at least two independent experiments.

### 4.7. Statistical Analysis

*Candida* growth curves were compared by ANOVA and Dunnett’s multiple comparisons test, *Candida* cell counts were compared by Kruskal-Wallis test. Statistical analysis was performed by using Prism 8.0.1 (GraphPad software, San Diego, CA, USA).

## 5. Conclusions

HA and LCFS from vaginal *L. crispatus* BC5 showed encouraging anti-*Candida* activity, which was also found towards *Candida* species that are generally resistant to conventional antifungals. The MCC-based vaginal matrix containing HA and LCFS represented a promising prototype for the treatment of VVC.

## Figures and Tables

**Table 1:** MIC (mg/mL) of HA and LCFS alone, and FICI values calculated on *Candida* growth inhibition data obtained by HA and LCFS combined treatment at 24 h.

*Candida* Isolate	MIC HA (mg/mL)	MIC LCFS (mg/mL)	FICI
*C. albicans* SO1	4	62.5	1
*C. albicans* SO2	4	62.5	0.75
*C. glabrata* SO17	4	62.5	1.5
*C. glabrata* SO18	1	62.5	1.25
*C. tropicalis* SO24	4	62.5	1.1
*C. krusei* SO26	4	125	2.1

**Table 2:** Viability of *C. albicans* SO1 in culture medium (SD broth) with and without MCC vaginal matrices containing HA and LCFS. MCC matrices without active molecules were used as control. Microbial concentration was assessed at the inoculum time (t = 0) and after 6 (t = 6 h) and 24 (t = 24 h) hours of incubation and expressed as mean CFU/mL ± standard deviation.

	Without Matrix	Matrix MCC Alone	Matrix MCC + HA + LCFS
t = 0	(1.60 ± 0.18) × 10^5^	(1.60 ± 0.18) × 10^5^	(1.60 ± 0.18) × 10^5^
t = 6 h	(1.52 ± 0.25) × 10^6^	(9.92 ± 1.69) × 10^5^	(1.66 ± 0.30) × 10^5^ *^,#^
t = 24 h	(3.18 ± 0.85) × 10^7^	(3.20 ± 0.23) × 10^7^	(5.92 ± 1.86) × 10^6^ *^,#^

* *p* < 0.005 vs. control without matrix, ^#^ *p* < 0.005 vs. MCC matrix.

## Data Availability

Data is contained within the article.
